# Cultural Aspects of Suicide

**DOI:** 10.1100/tsw.2005.88

**Published:** 2005-09-08

**Authors:** Hari D. Maharajh, Petal S. Abdool

**Affiliations:** Department of Medical Sciences, University of West Indies, MT Hope, West Indies, Jamaica

**Keywords:** Human development, suicide, public health, Trinidad

## Abstract

Undefined cultural factors cannot be dismissed and significantly contribute to the worldwide incidence of death by suicide. Culture is an all embracing term and defines the relationship of an individual to his environment. This study seeks to investigate the effect of culture on suicide both regionally and internationally. Culture-bound syndrome with suicidal behaviours specific to a particular culture or geographical region are discussed. Opinions are divided as to the status of religious martyrs. The law itself is silent on many aspects of suicidal behaviour and despite decriminalization of suicide as self-murder, the latter remains on the statutes of many developing countries. The Caribbean region is of concern due to its steady rise in mean suicide rate, especially in Trinidad and Tobago where socio-cultural factors are instrumental in influencing suicidal behaviour. These include transgenerational cultural conflicts, psycho-social problems, media exposure, unemployment, social distress, religion and family structure. The methods used are attributed to accessibility and lethality. Ingestion of poisonous substances is most popular followed by hanging. The gender differences seen with regard to suicidality can also be attributed to gender related psychopathology and psychosocial differences in help-seeking behaviour. These are influenced by the cultural environment to which the individual is exposed. Culture provides coping strategies to individuals; as civilization advances many of these coping mechanisms are lost unclothing the genetic predisposition of vulnerable groups. In the management of suicidal behaviour, a system of therapeutic re-culturation is needed with an emphasis on relevant culture- based therapies.

## INTRODUCTION

The effect of culture on human behaviour is equivocal with opposing views of both protective and destructive tendencies. Culture may provide a support system to an individual's vulnerability and defences related to ego-functioning, or on the other hand,, may perpetuate an ecologically unhealthy environment. Often,the transgenerational loss of the old culture will result in conflicts between the mores of the traditional culture and the expectations of the modernizing society. Thus, renunciation of the old culture without assimilation of the new predisposes individuals to behavioural disturbances such as suicide.

Suicidal behaviour with completed suicide is one of the leading causes of premature death[1]. This high prevalence has resulted in a growing desire to elucidate the underlying or associated factors. This study seeks to review suicidal behaviours across cultures with special reference to ethnicity, religion, lifestyles, legislation and methods used in Trinidad and Tobago. Within the Caribbean region, the issue of suicidal behaviour is a growing problem. According to the World Health Organization, Trinidad and Tobago have the second highest rate of suicide in all the West Indian islands[2]. Very few of these islands have conducted community surveys with an emphasis on causation and prevention.

Currently, there exist few cross-national comparisons of the rates of suicidal ideation and attempts across diverse culture and countries. Interestingly, one US publication involving nine independently conducted epidemiological surveys that utilized similar diagnostic assessment and criteria provided an opportunity to obtain that data. This was possible since it was performed on over 40,000 subjects drawn from the US, Canada, Puerto Rico, France, West Germany, Lebanon, Taiwan, Korea and New Zealand. Females when compared to males had only marginally higher rates of suicidal ideations in most countries, but this reached a two-fold increase in Taiwan. The rates of suicidal ideation varied widely in each country and attempts to explain these variations led to the conclusion that cultural factors which are not yet clearly defined may be partly responsible[3].

Therefore, the impact of culture on suicide is one which cannot be dismissed and this relationship needs further evaluation if we hope to decrease the escalating numbers of suicides and attempted suicides across the globe.

## CULTURE AND MENTAL HEALTH

The influences of cultural behaviours on mental well-being have been an area of long and intensive investigations[4-6] and remain an enigma even today. In the past, social scientists have interpreted disorders of the mind as a form of social deviancy where the individual was regarded as mentally ill because he had broken the local codes of social conduct[7]. These concepts of psychiatric disorders as a deviation from social and cultural norms rather than a product of an underlying biological dysfunction have not been accepted by many psychiatrists. Many are of the opinion that social impairment is insufficient and psychiatric diagnosis should be made only on the basis of mental status, not in terms of cultural behaviour.

Cultural behaviours can be defined as an adaptive response occurring in organized social groups whose lifestyles, sentiments and interactions have been transgenerationally transmitted. These cultural factors determine behaviour and through a system of ego-protective defense mechanisms seek to explain an individual’s relationship with his environment. These behaviours are often associated with compensatory defenses which are sometimes conceptualized and socially contrived in the context of native humour[8].

## CULTURE-BOUND SYNDROMES

Culture-bound syndromes are current locality-specific patterns of aberrant behaviour and troubling experiences that may or may not be linked to a particular DSM-IV-TR diagnostic category. Many of these patterns are indigenously considered to be illnesses or at least afflictions and most have local names[9]. Culture-bound syndromes are thus culturally determined abnormal behaviour patterns that are specific to a particular culture or geographical region. These behaviours express core cultural themes and have a wide range of symbolic meanings – social, moral and psychological[10].

Genuine culture-bound syndromes are not exclusively linked to a particular culture, but rather are related to a prominent cultural emphasis or to a specific social stress situation[11,12]. Syndromes have been described with a cultural emphasis on a number of themes. Some are related to suicidal behaviour such as amok in south east Asia and Malaysia[13-15], tabanca in Trinidad[16] and Hi-Wa-itck in Mohave American Indian[17].

Over the past two decades, Trinidad and Tobago, resplendent in its cultural diversity, has attracted a number of visiting researchers. Littlewood[16] has described Tabanca (lovesick behaviour) as an indigenous conceptualization of depression in rural Trinidad that leads to suicide. This, he claims, is an affliction of working class Afro-Caribbean males who aspire to white and middle class values and lifestyles. His views are considered to be a misinterpretation of this cultural phenomenon. Local psychiatrists[18,19] have been critical of his findings stating that he did not take into account the cultural milieu that colours expression of the behaviour and that he was blinkered by his own unconscious cultural assumptions. Similarly, Eriksen[20] has described liming in Trinidad as a dignified art of doing nothing, noting that one cannot be recognized as a real Trinidadian unless one masters the art of doing nothing. This descriptive transcendence into anomie of an entire population underlines the need for local input in the interpretation of socio-cultural phenomena.

## LAW, CULTURE AND SUICIDE

Culture is an all-embracing term and incorporates input from both ethnicity and race. Ethnicity refers to selected cultural and physical features of groups varying in language, religion, tradition, food, dress, skin colour and body morphology. Race refers to a biological subspecies with distinct phenotypes, but racism has moved to the social domain and appears to be based on issues of power and political control rather than biological superiority. Mention has already been made of culture-bound syndromes with suicidal behaviour which are unconscious acts resulting in death. These acts, in the true meaning of the word suicide, cannot be classified as deliberate acts of self-annihilation. Suicidal behaviour is an illegal act and this criminal offence must have the elements of *actus reus* which denotes a guilty act and *mens rea* which denotes a guilty mind. Suicide remains a criminal act in the Caribbean region, but has been decriminalized in many countries. Suicide is no longer considered to be self-murder or an offence. Legally, a person’s life belongs to the state and not to himself and he is, therefore, forbidden from bodily harming himself as he cannot others. Suicide attempters are not charged, but deemed to be of unsound mind and must be assessed by a psychiatrist. In complete suicide, the coroner’s verdict is based on the following[21]:

- The death must be deemed as unnatural
- The perpetrator of the course of action that resulted in death has to be determined as the deceased himself.
- Motive for self-destruction has to be established.

In the defence of a survivor of a suicidal pact, since he caused the death of another deliberately and is prima facie guilty of murdering him, he must prove that there was an agreement that they should both die and when he committed the act, he himself was under the settled expectation of dying.

The strict legal approach does not take into consideration the diverse cultural beliefs of populations and results in census inaccuracies. It is well known that there are culturally sanctioned forms of self-annihilation. For example, it is acceptable in the Japanese culture to commit suicide if you have lost face. This however is not coded as suicide. Another contemporary problem is that of suicide bombers and altruistic suicides. Research suggests that they may not be different from other suicides[22]. If this is indeed the case then more evidence is needed to elucidate the psychological aspects behind these acts.

## SUICIDE ACROSS CULTURES

While culture remains an important determinant in the patterns of suicide worldwide, loss of cultural emphasis due to the superimposition of western values, a reactive cultural revolution back to fundamentalist principles, social and political upheavals and road maps of geographical possession have all influenced suicide rates. These rapidly changing variables over time make it difficult to compare rates across cultures since they are often contextual. Instead, suicidal patterns across various cultures will be reviewed here with references to ethnic variations and religious practices.

The close relationship between culture and ethnicity makes discussion of one without the other almost impossible. A study from the United States in Fulton County examined ethnic difference in patterns of suicide across the life cycle and found that African Americans commit suicide at rates much lower than those for whites. The former group do so at a younger age group and have a narrow, age-defined window of vulnerability which has been ascribed to certain unidentified protective factors and age-specific psychopathological processes[23].

In many Moslem countries, low rates of suicide are reported. This may be the result of strict legal and religious sanctions against suicidal behaviour. In Pakistan, very low rates of suicide are reported and this may be misleading due to underreporting[24]. Culture, therefore, may sometimes affect the reporting rather than the suicidal act. Suicide is reportedly low in the Jewish population, however, Lubin et al[25] have reported higher suicide rates in Israeli Jewish males and females than Arab males and females. The effect of lifestyles, immigration, military service, absence of peace and media exposure to violence and contagion need further study in that country.

Latinos, or Latin Americans, appear to be relatively protected against suicidal behaviour and some of these protective factors are thought to be related to cultural constructs that provide a buffer against suicidal behaviour[26]. One study used the Reasons for Living Inventory (RFLI) in order to capture protective factors against suicidal behaviour in Latinos and non-Latinos. The results showed that being Latino was independently associated with less suicidal ideations, while the decrease in other suicidal behaviours was attributed to a stronger relationship to moral objections to suicide and coping skills[26].

During the period 1830-1920, there was the export of Indian labour overseas to Fiji, Malaysia, Ceylon, Mauritius, Burma, Uganda, South Africa and the Caribbean[27]. High rates of suicide have been reported for immigrant groups of East Indians for all generations throughout the world. These rates are in comparison to the indigenous populations in the new environment rather than that of the sending country.

In Fiji, higher rates among East Indians were associated with rurality, biocides, religious and cultural factors[28]. Although the incidence of suicide in Malaysia was relatively low, forty nine percent of all suicides were ethnic Indians who constituted only eight percent of the Malaysian population, 38% suicide rates were among Chinese who formed 26% of the population and 3.6 % were Malay that represented 59 % of the population[29]. Similarly, in Singapore, Indians had the highest suicide rates (19.3 per 100,000) compared to the other ethnic groups of Chinese 16.2 per 100,000 and Malays (2.3 per 100,000)[30].

Some studies from Hong Kong have reported lower rates of suicide among adolescents aged 10-24 years than the adult population[31,32], while others have revealed a significant increase in the suicide rates among adolescents aged 15 to 24 years in the 1980s[33]. These authors have postulated protective cultural factors that inhibit suicidal behaviour, since killing or hurting oneself is regarded as an unfilial act in Confucian teaching and suicidal behaviour itself is discouraged in the Buddhist and Taoist philosophies. Suicidality has been related to inwardly directed aggression. Aubert et al[34] looked at the prevalence of suicidal ideations and behaviours in Canadian students of Chinese and non-Chinese origin. While the Chinese Canadians had higher levels of suicidality and hostility, the actual incidence of aggressive behaviours directed toward self or others was less frequent. These results were attributed to the influence of Chinese culture on emotional restraint especially with regards to aggressiveness[34].

Suicidal behaviour has been reported as being high in East Indian women. Young Fijian Indian women[28] and young South African Indian women have high rates of suicidal behaviour[35]. In these multi-ethnic societies, not unlike Trinidad, women are caught between the western modernizing culture and their own traditional home culture and often have difficulties in conceptualizing to which culture they belong. Adoption of western values with a shifting of traditional role definitions results in a borderline cultural state which serves as a precursor for marital dysfunction, emotional disorders and suicidal behaviour. Culture and religion are protective factors in suicidal behaviour[1].

In Saudi Arabia, despite its economic prosperity and development, suicide rates are low in the native population. In one study, immigrants accounted for 77% of all cases with East Indians accounting for 43%[36]. High suicide rates were also found among young Asian women, 20% of which were by burning[37]. Suicide ratios were significantly low in Bangladeshi , Sri Lankan and Pakistani born men at all ages, but higher in young Indian and East African men[37].

## SUICIDE IN THE CARIBBEAN REGION

Suicide rates among Caribbean countries are variable with a clear cut trend of male predominance and higher incidences in those islands with a multi-ethnic population. The WHO rates listed below are ten years or more old and it is expected that over this period rates have changed (See Table 1).

**Table 1.**
**Suicide rates per 100,000 in the Caribbean Islands (WHO 2002)**

| Country | Year | Total Suicide | Male Suicide | Female Suicide |
|---|---|---|---|---|
| Bahamas | 1995 | 1.1 | 2.2 | 0 |
| Barbados | 1995 | 6.5 | 9.5 | 3.7 |
| Cuba | 1995 | 18.3 | 24.5 | 12.0 |
| Guyana | 1994 | 10.5 | 14.6 | 6.5 |
| Jamaica | 1985 | 0.3 | 0.5 | 0.2 |
| St. Lucia | 1988 | 7.5 | 9.3 | 5.8 |
| Trinidad and Tobago | 1994 | 11.6 | 17.9 | 5.0 |

In a more recent study in Trinidad and Tobago, the overall mean suicide rate calculated for the eight year period 1990-1997 was found to be 20.6/100,000 for the male population and 5.4/100,000 for the female population. For males, two peaks were found at the 25 to 34 and 55 to 64 age groups, and for females at the 15 to 24 and over 65 age groups[38]. In Trinidad and Tobago, adolescent suicide rate was 2.3 times more common in males than females in the 15 to 24 age groups and overall rates were approximately 3.5 to 4 times more common in males than females.

In an analysis of 1,845 respondents aged 14 to 20 years from 24 schools across Trinidad and Tobago, a number of predictors of suicidal behaviours were identified. Attendance to a religious institution was found to low suicidal ideation, while prayer with the family altered both suicidal ideations and attempts. Other factors identified were gender, family structure and alcohol abuse[1]. The Indian family system was perceived as being suicide prone with the dynamics of its family structure being a contributory factor. Individuals with alcohol abuse present in the family had a two fold increase in suicidal behaviour. Research in Trinidad has reported alcoholism being highest among the East Indian population[39,40].

Indian adolescents were more likely to report alcohol abuse in the family than the other races. In Trinidad and Tobago, ethnic differences were evident in current suicidal ideation with Indian adolescents having higher mean scores than African adolescents[1].

A number of reasons have been proffered for the high suicide rates amongst Indo-Trinidadians:

## Transgenerational Cultural Conflicts with Family Culpability [41,42]

The following case history highlights a common occurrence of dying with dignity. A 20 year old Indo-Trinidadian University student from an Orthodox Hindu background was seen in consultation following the ingestion of a few teaspoonfuls of a poisonous substance. After clinical observations of the blistering of her lips, mouth and throat, laboratory and family investigations revealed that she had drunk gramoxone (paraquat), a deadly biocide. According to her room-mates, during her first year at University she began “hanging out with the wrong crowd and was shunne”. Unknown to her parents, she had established a relationship with an Afro-Caribbean male student, neglected her work, allegedly drank heavily and failed her first year. Her father, on receiving news of her behaviour, was furious and verbally abused her for bringing shame and disgrace to his family. In a fit of temper, he implied that her death may return some dignity to the family’s name and that “she was better off dead since she had shamed the family”. That evening she took the fatal weedicide. Findings on psychological autopsy revealed that the father felt little guilt and had poor insight into his culpability. He justified his actions by acting in accordance with the Hindu religious text that denounces the intermixture of caste. Purity, avoidance of shame and contamination and codes of behaviour are entrenched in the teachings of the holy Hindu texts.

## Psych-social Problems

Lover’s quarrels, interpersonal problems, religious beliefs, cultural rigidity, marginalization, ethnic disadvantage, poverty, family problems and depression have been stated as precipitating causes of suicidal behaviour among Indo-Trinidadians[43]. In addition, when compared to Afro-Trinidadians, Indians suffered more from physical abuse and unwanted pregnancy[1]. The two major races in Trinidad and Tobago are Indo-Trinidadians (40.3%) and Afro-Trinidadians (39.6%). The government is African based and Indo-Trinidadians are faced with discrimination in position and promotion, housing, unequal educational opportunities, as for example in obtaining government scholarships or placement in training programmes. Crime directed against the Indo-Trinidadian community is on the increase with young Indo-Trinidadian businessmen being kidnapped for ransom. The Principle of Fairness Committee founded in 2004 recently held a meeting at the University of the West Indies and warned that Trinidad and Tobago was nearing collapse because of racial division, crime and improper governance by political leaders. They called for constitutional reform[44].

Here is another example of stress on the job. A 49 year old civil servant in a Government’s Ministry complained of a lack of satisfaction at the workplace and his inability to cope with the administration at the office. He was an extremely diligent worker who spent extra hours every evening “in clearing his desk”. Despite his seniority in the public service and periods of acting as head of his division, he was always denied the permanent position as director. On two previous occasions, people junior to him were promoted as his supervisors and he was asked to train them for their positions. According to him, he did all the work, while they held the senior positions and did nothing. He was denied vacation leave when he applied, received bad reports from his supervisors and felt marginalized in the office since he belonged to a different ethnic grouping than other workers who openly made references “about his kind of people”. A few weeks after he was denied promotion for the third time, he began drinking heavily. He hardly turned up for work and his physical health deteriorated. He was admitted to an Alcohol Rehabilitation Centre where he spent six weeks. He improved well, but relapsed on returning to work two months later. He continued missing work and sliding downhill, refusing further treatment. One year later, he was found dead in bed with an empty bottle of a toxic substance on the carpet. On psychological autopsy, his family blamed the lack of equal opportunity, marginalization and ethnic disadvantage as the cause of his death.

## Media Reporting of Suicides

There are no guidelines for the reporting of suicides in Trindad and other Caribbean countries. These are often sensationalized with graphic descriptions in headline coverage. There is the depiction of live televised suicides and homicides. This is contributory to the high suicidal rates[41]. Another contributory factor may be the viewing of popular Hindi films that often depict maladaptive methods of handling personal, family and stressful situations. There seems to be a need for control.

## Unemployment and Social Distress

Researchers in Trinidad have identified unemployment as a possible factor of increased rates of suicide. An association has been established between social distress and suicidal behaviour[45]. Preliminary findings in an ongoing study have shown no relationship between suicide and employment in Trinidad and Tobago for the period 1998-2002.

## Religion and Suicide

In 1879, Morselli reported decreased rates of suicide among Catholics when compared to Protestants and Jews[46]. Subsequently, religious persuasions have been associated with suicidal behaviour with higher rates being found among Hindus in Trinidad[1] and in Suriname[47]. Islam has strong religious sanctions against suicide and it is therefore expected that suicide rates will be low. In Judaism, Islam and Christianity to take one’s life is considered a transgression against God’s law. The Judeo-Christian teachings disapproved of the burial of suicide victims according to the Talmud (Mishnah). Death by suicide prevented the individual from eternal happiness. Hinduism is more tolerant of suicide, but has prescriptions for the wandering of the soul in disruptions of the cycles of birth and death in reincarnation. Ritualistic suicide of *suttee*, now illegal, allowed the wife to throw herself into her husband’s funeral pyre. The early Christian voluntary martyrdom is now being adopted by followers of Islam. It seems as though religion has a meaningful part to play in the lives of individuals and is associated with socially acceptable behaviour.

## Family Structure

Most Indo-Trinidadians live in extended families, or nuclear families, that are in close contact to the extended families. The family structure is patriarchal with the father being the provider of the basic needs of culture, education, religion and discipline. The African family is patterned in a matriarchal model, unstable with multiple relationships and absent fathers. Indo-Trinidadian families with extended collective supervision of adolescents and young adults result in more family quarrels, conflicts and acting out behaviour. Depression and alcohol abuse that had its origin during the indentureship period in the sugar plantations are common findings in the Indian family. These are well known precursors of suicidal behaviour.

## METHODS USED

The methods used in suicidal acts are closely equated to the mores, occupation, educational and economic development and accessibility of lethal substances or weapons in that society. Suicidal victims are not usually creative and have a tendency to use tested methods in everyday use.

In Trinidad and Tobago, the commonest method of death by suicide is self-poisoning caused by the ingestion of a deadly weedicide known as paraquat or gramoxone, a member of the organophosphate group of compounds. It is popularly used by farmers in the rural agricultural regions and can be purchased by anyone. There is no legislation with respect to purchasing the compound or storage at home.

Forty years ago, death by hanging was the most used method of suicide followed by drowning. Again, in rural areas, those associated with farming strangled themselves with rope that was utilized for tethering animals. The rope was first smoothened with candle wax to facilitate easy movement and tied to branches of trees or the beams and ceilings of dwelling houses. The other end was made into a noose and placed around the neck. Death was quick due to strangulation or in some cases through broken necks due to jumping. This method was more common among males, while females preferred drowning. Hanging is still the second most popular form of suicide.

Over the last five years, there has been an increase of suicide by gunshot wounds. This is due to the high level of crime within the country and the inability of the authorities to control the distribution and possession of illegal firearms within society. Jumping is uncommon and may be due to the absence of high rise buildings. Asphyxiation through carbon monoxide poisoning is about 1-2 per million and when reported often involves suicidal pacts of lovers in motor vehicles. Rare cases of auto-erotic asphyxiation have been reported as well as locking oneself into a deep-freeze refrigerator. Self immolation by fire is unknown. Suspected suicidal death through motor vehicular accidents, voluntary drowning or fire-setting to self occupied buildings are difficult to establish.

Among adolescents the commonest form of suicidal attempts is through prescription drugs overdose. Within recent times parasuicidal attempts of substance intoxication are becoming more common. Self–mutilation with wrists cutting and body carvings are on the rise. In this group, daring drug induced behaviour can result in fatal outcomes such as drowning, jumping or accidents.

## DISCUSSION

Culture is dynamic, it is ever changing. Contemporary human behaviour is influenced by political, economic, social, technological and cultural factors. These influences have affected the most primitive societies and are not always beneficial. Lambo[48] noted that in many parts of Africa, social and cultural changes led to altered social habits, to economic pressures and the relaxation of the traditional authority. The changes in the structure and function of the traditional African family have resulted in higher incidences of behavioural disturbances such as drug addiction, abnormal sexuality, delinquency and other antisocial behaviour.

Valid distinctions are made between culture and culture bound-syndromes. The most significant is that the latter involved aberrant behaviours that are specific to particular regions or group of people.

The role of the law in defining suicide and the inherent short-comings in such a definition is a prime example of the need for further consideration of the impact of culture on suicidal behaviour. Once this is firmly established it may dispel any misconceptions and census inaccuracies. It may also lead to new legislation with regard to suicide bombers and altruistic suicides.

Recent studies have classified suicide bombers within the same group as conventional suicides. This however may be an instance where strongly held cultural and religious beliefs help motivate their decision to commit suicide. In the Islamic faith, the suicide bomber is upheld as a hero (Shahid or martyr)

by those who support the cause. “Hamas”, or rewards of the afterlife, may serve as additional impetus[49]. Coercion and deception may be other factors that are not relevant in other cases.

The above findings indicate that suicidal ideation and behaviour vary from nation to nation and across cultures. The latter statement assumes that ethnicity and religion can be used as a means of assessing various cultures. It is therefore evident from the research that some cultures appear to be more suicide prone than others. Religious beliefs, cultural norms endorsed by certain groups and the family structure can act as buffers for suicidal behaviour[26]. A good example of this is the explanation given for the lower rates of suicide among adolescents in Hong Kong when compared to Western nations[31]. In other cases however, ethnicity and religion as seen in the Hindu (East Indian) population may inculcate maladaptive coping styles that serve as a foundation for suicidal behaviour. A genetic predisposition to alcoholism and a turbulent family structure are considered to be risk factors for suicidality[1]. An essential consideration when reviewing suicide patterns across cultures is the propensity for under-reporting that is indigenous to cultures that do not sanction suicidal behaviour.

The gender differences seen with regard to suicidality can also be attributed to gender related psychopathology and psychosocial differences in help-seeking behaviour[50,51]. The latter are all influenced by the cultural environment to which the individual is exposed. One common factor that is evident cross culturally is the clear cut trend of male predominance.

The Caribbean region is an area that is cause for growing concern due to its steady rise in overall mean suicide rate. Trinidad and Tobago was particularly highlighted, since studies showed many socio-cultural factors were instrumental in influencing suicidal behaviour. These factors included transgenerational cultural conflicts, psycho-social problems, media exposure, unemployment, social distress, religion and family structure.

Analysis of the methods used revealed a common trend across some cultures. This was attributed to accessibility and lethality. Ingestion of poisonous substances was most popular followed by hanging.

## CONCLUSIONS

A great deal of research needs to be performed on a global scale to ascertain the degree to which culture impacts on suicidality and this in turn can be used to address the rising rates of these self-destructive behaviours. Culture provides coping strategies for individuals. As civilization advances many of these coping mechanisms are lost, unclothing the genetic predisposition of vulnerable groups. In the management of suicidal behaviour, a system of therapeutic re-culturation is needed with an emphasis on ethnohistiography, psycho-spiritual and culture therapy.

## BIOSKETCHES

**Hari D Maharajh,** BSc (Hons), MBBS (UWI), MRCPsych, Dip Clin Neuro, LLB (Hons), CMT; FRCPsych, Consultant Neuropsychiatrist, is a Senior Lecturer in the Department of Clinical Medicine at the University of the West Indies, Mount Hope, Trinidad. He has pursued an academic career with interests in medicine, law and social sciences. He studied zoology and chemistry at the University of Manitoba, medicine at the University of The West Indies, psychiatry at St. George's Hospital, and neurology at the Institute of Neurology, London. He also read law at the University of London. In 2000 he was awarded the Chaconia Gold Metal for community service. He has published a number of scientific and popular articles and is the author of a textbook in neurology. He enjoys teaching, writing and doing research in adolescents and cultural issues. E-mail: drharim@carib-link.net

**Petal S. Abdool,** MBBS is a senior House Officer in the Psychiatry Department of the San Fernando General Hospital located in Trinidad and Tobago. She is a graduate of the University of the West Indies, Faculty of Medical Sciences Eric Williams Complex Mount Hope. Currently, she is pursuing post-graduate studies in psychiatry at the aforementioned university. A national scholarship winner with a keen interest in research, she enjoys reading, squash and public speaking. E-mail: petal_abdool@yahoo.co.uk.
